# Long-term safety and clinical outcomes of olipudase alfa enzyme replacement therapy in pediatric patients with acid sphingomyelinase deficiency: two-year results

**DOI:** 10.1186/s13023-022-02587-0

**Published:** 2022-12-14

**Authors:** George A. Diaz, Roberto Giugliani, Nathalie Guffon, Simon A. Jones, Eugen Mengel, Maurizio Scarpa, Peter Witters, Abhimanyu Yarramaneni, Jing Li, Nicole M. Armstrong, Yong Kim, Catherine Ortemann-Renon, Monica Kumar

**Affiliations:** 1 Department of Genetics and Genomic Sciences, Icahn School of Medicine at Mount Sinai, 1 Gustave L. Levy Place, New York, NY 10029 USA; 2 Medical Genetics Service HCPA, Department of Genetics UFRGS, DASA and Casa dos Raros, Porto Alegre, Brazil; 3 Reference Centre of Inherited Metabolic Disease in Femme Mère Enfant Hospital, Hospices Civils of Lyon, Lyon, France; 4 Manchester University National Health Service Trust, St Mary’s Hospital, Manchester, UK; 5 Institute of Clinical Science for Lysosomal Storage Disorders, SphinCS GmbH, Mainz, Germany; 6 University Hospital of Udine, Udine, Italy; 7 University Hospitals Leuven, Leuven, Belgium; 8 Sanofi, Bridgewater, NJ USA; 9 Sanofi, Cambridge, MA USA; 10 Sanofi, Paris, France

**Keywords:** Recombinant human acid sphingomyelinase, Dose escalation, Organomegaly, Lung diffusing capacity, Acid sphingomyelinase deficiency, Niemann–Pick type B

## Abstract

**Background:**

Olipudase alfa is a recombinant human acid sphingomyelinase (ASM) enzyme replacement therapy (ERT) for non-central-nervous-system manifestations of acid sphingomyelinase deficiency (ASMD). We report 2-year cumulative safety and efficacy data after olipudase alfa treatment in 20 children (four adolescents [12–17 year], nine children [6–11 year], and seven infants/early child [1–5 year]) with baseline splenomegaly and growth deficits who completed the 1-year ASCEND-Peds clinical trial (NCT02292654) and who continue to receive olipudase alfa in a long-term study (NCT02004704). Efficacy endpoints include spleen and liver volumes, diffusing capacity of the lung for carbon monoxide (DL_CO_), high-resolution computed tomography (HRCT) lung imaging, lipid profiles, liver function tests, and height Z-scores.

**Results:**

All 20 former ASCEND-Peds patients completed at least 2 years of olipudase alfa treatment. No patient discontinued and no new safety issue arose during the second year of treatment; 99% of adverse events were mild or moderate. During year 2, one patient had two treatment-related serious events of hypersensitivity that resolved. Mean reductions from baseline in spleen and liver volumes were 61% and 49%, respectively (*p* < 0.0001) and mean percent-predicted-DL_CO_ increased by 46.6% (*p* < 0.0001) in nine patients who performed the test at baseline. Lipid profiles and elevated liver transaminase levels that improved or normalized by 1 year remained stable. Mean height Z-scores improved in all age groups (mean change from baseline 1.17, *P* < 0.0001).

**Conclusion:**

Olipudase alfa was generally well-tolerated during 2 years of treatment. Improvements in clinically relevant disease endpoints observed during the first year of treatment were maintained or augmented in the second year.

*Trial registration* NCT02004704 registered 26 Nov 2013, https://clinicaltrials.gov/ct2/show/record/NCT02004704.

**Supplementary Information:**

The online version contains supplementary material available at 10.1186/s13023-022-02587-0.

## Background

Acid sphingomyelinase deficiency (ASMD) (historically known as Niemann-Pick disease [NPD] types A [OMIM257200], B [OMIM607616], A/B) is an inherited lysosomal storage disease characterized by deficient activity of the enzyme acid sphingomyelinase (ASM). Deficiency of ASM results in accumulation of glycosphingolipids, including the primary substrate sphingomyelin, in tissue macrophages and hepatocytes, and in the most severe form of ASMD, in neurons [1, 2]. Manifestations include hepatosplenomegaly and interstitial lung disease (ILD) accompanied by thrombocytopenia, bleeding/bruising, dyslipidemia, growth deficits and delayed puberty, osteoporosis/osteopenia, liver dysfunction with progressive fibrosis, and cardiac disease [3, 4]. ASMD subtypes reflect a disease spectrum ranging from ASMD type A (infantile neurovisceral disease) [5] that is uniformly fatal within the first 3 years of childhood, to chronic forms ASMD type B (chronic visceral) and ASMD type A/B (chronic neurovisceral) that lack predominant neurodegeneration and frequently present with visceral symptoms in childhood. Chronic forms of ASMD are associated with significant morbidity and sometimes early mortality due to respiratory or liver failure [6–9]. Supportive care is used for symptom management in the absence of an approved disease-modifying therapy [10].

Olipudase alfa (Xenpozyme®) is a recombinant human ASM enzyme replacement therapy (ERT) approved for the treatment of the non-central nervous system manifestations of ASMD in children and adults. Olipudase alfa is well-tolerated in adults [11–13] and pediatric [14] patients with chronic ASMD after a within-patient dose escalation regimen designed to gradually debulk tissue sphingomyelin [15, 16]. Olipudase alfa was associated with clinically significant improvements in disease pathology and multiple endpoints compared with placebo in adults with ASMD [13]. In pediatric patients with chronic ASMD, an open label single-arm study demonstrated improvements across a range of endpoints after 1 year of treatment [14]. This paper reports on the safety and clinical outcomes data for these pediatric patients ranging in age from infancy to adolescence who have received olipudase alfa infusions every 2 weeks for 24 months.

## Methods

### Study design/participants

As described previously, 20 pediatric patients with confirmed ASMD, spleen volume ≥ 5 multiples of normal (MN), and height Z-score ≤ − 1 participated in the open-label, single arm ASCEND-Peds trial [14]. The study included patients with ASMD types B or A/B and no patients with ASMD type A were enrolled. After the 52-week (efficacy)/64-week (safety) primary analysis periods, all 20 patients have continued olipudase alfa treatment in a long-term study (trial registration NCT02004704; EudraCT2013-000051-40). Institutional Review Boards at study sites approved the protocol and patients/parents provided written consent.

### Olipudase alfa infusions

Olipudase alfa infusions were administered every 2 weeks. All patients underwent individualized dose escalation over a minimum of 16 weeks in the ASCEND-Peds trial and achieved the target maintenance dose of 3 mg/kg as described previously [14].

### Outcomes

All safety and efficacy outcomes represent cumulative patient data through 24 months from olipudase alfa initiation. Adverse events were summarized by incidence, seriousness, severity, and relationship to olipudase alfa treatment. Clinical parameters (spleen volume and liver volume in multiples of normal [MN], where normal spleen and liver volumes are considered to be 0.2% and 2.5% of body weight, respectively [17], percent predicted diffusing capacity of the lung for carbon monoxide [DL_CO_] adjusted for hemoglobin, volumetric lung function tests, high-resolution computed tomography [HRCT] lung imaging scores, platelet counts, liver function, plasma lipid profiles, height Z-scores) and biomarkers reflecting the activity of ASMD (plasma chitotriosidase activity [normalized, µmol/L/hr], plasma lyso-sphingomyelin [μg/L]) were evaluated as described previously [14]. X-ray of the left hand, fingers and wrist were used to determine bone age compared to actual age using the Greulich and Pyle atlas [18].

### Analyses

Analyses were performed overall and by baseline age group. Descriptive statistics with standard deviations were used for continuous variables and for concentration–time data. Categorical variables were summarized using frequencies and percents. Change and percent change from baseline were analyzed with the analysis of covariance (ANCOVA) method adjusting for baseline value and without multiplicity adjustment except for HRCT scores, for which a linear regression model was used to assess change from baseline adjusted by baseline HRCT scores. *P* values from the various tests are presented for the mean or least square (LS) mean change (or percent change) from baseline to 24 months, and all are nominal.

## Results

### Patient baseline characteristics

**Fig. 1:**
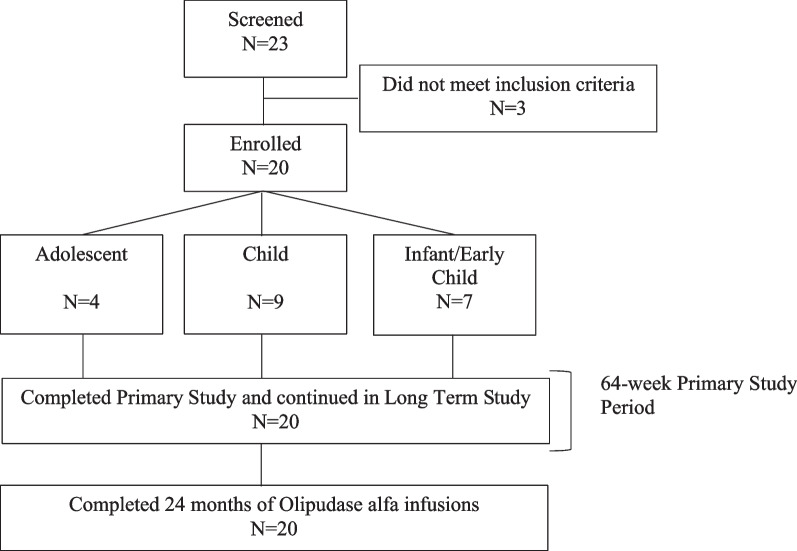
Patient disposition

Twenty enrolled patients completed the primary analysis period of the ASCEND-Peds trial and continued olipudase alfa treatment in the long-term study (Fig. 1). Baseline characteristics have been previously published [14] and are shown in Additional file 1: Table S1. Age at symptom onset ranged from 1.9 months to 3.9 years and presenting symptoms at disease onset included splenomegaly (90%), hepatomegaly (90%), respiratory diseases (35%), thrombocytopenia (25%) and excessive bleeding and bruising (10%). Age at trial enrollment ranged from 1 to 17 years, males and females were equally represented, and the majority were Caucasian. At baseline, 12 patients (60%) had severe splenomegaly (> 15 MN).

### Olipudase alfa dosing

The median time (min, max) to achieve the target dose during the primary ASCEND-Peds 64-week study was 18 weeks (16, 50). All patients maintained the target dose of 3 mg/kg during the second year of treatment. One patient had a temporary dose reduction during week 67 of treatment due to adverse events of sore throat, pharyngotonsillitis, bronchitis, and fever unrelated to olipudase alfa treatment.

### Safety

There were no permanent treatment discontinuations or withdrawals from the study during 24 months of olipudase alfa treatment. Treatment-emergent adverse events occurring during the primary 64-week safety period have been described in detail previously [14]. No new safety issues occurred during the second year of olipudase alfa treatment and 99% (1070/1077) of all treatment-emergent adverse events from first dose through month 24 were reported as mild (960/1077, 89%) or moderate (110/1077, 10%).

**Fig. 2:**
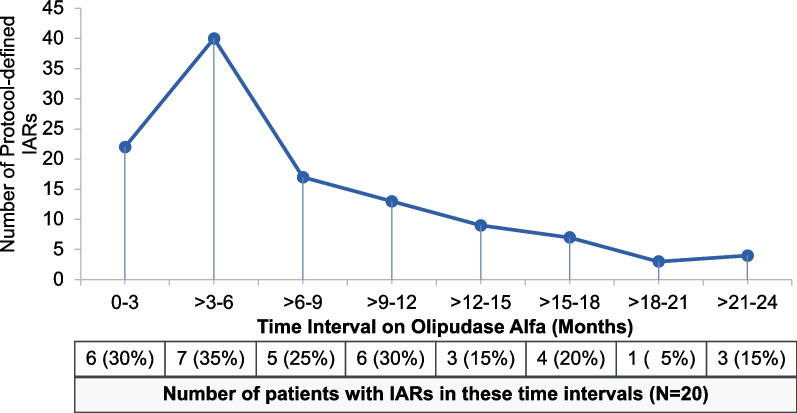
Time course of infusion-associated reactions (IARs) during olipudase alfa treatment

As reported for the primary safety period [14], the most common adverse events considered related to olipudase alfa were pyrexia, vomiting, urticaria, and headache, most of which were mild infusion-associated reactions (IARs) as shown in Table 1. IARs were reported for 13 patients during 24 months of olipudase alfa infusions. The number of IARs and number of patients with IARs peaked by 6 months and subsequently steadily decreased over time as shown in Fig. 2. Among 115 IAR events occurring from first dose, 99 (86%) were reported as mild, 15 (13%) as moderate and 1 (0.9%) as severe (an anaphylactic reaction in a patient during the second month of treatment as previously reported [14]). Among IARs occurring in the second year of treatment, none were severe, 19/23 (83%) were reported as mild, and 4/23 (17%) were reported as moderate.

**Table 1:** Related adverse events occurring in two or more patients during 24 months of olipudase alfa treatment

Related adverse event	Patients n (%) N=20	Events	Events categorized as IARs	Severity by event
Pyrexia	8 (40.0)	25	23/25	All mild
Vomiting	7 (35.0)	17	16/17	16 mild, 1 moderate
Urticaria	6 (30.0)	28	28/28	23 mild, 5 moderate
Headache	5 (25.0)	10	7/10	9 mild, 1 moderate
C-reactive protein increased	4 (20.0)	4	4/4	3 mild, 1 moderate
Nausea	4 (20.0)	4	4/4	2 mild, 2 moderate
Rash	3 (15.0)	6	4/6	5 mild, 1 moderate
Serum ferritin increased	3 (15.0)	3	3/3	2 mild, 1 moderate
Abdominal pain	2 (10.0)	6	5/6	All mild
Blood bilirubin increased	2 (10.0)	2	1/2	All mild
Erythema	2 (10.0)	3	3/3	All mild
Macule	2 (10.0)	3	0/3	All mild

*IAR* infusion-associated reaction

In addition to three patients with serious adverse events related to treatment that were reported during the 64-week primary analysis period and described previously [14], a 6-year-old patient had two serious adverse events consisting of IARs of mild hypersensitivity reactions (sneezing, flushing, facial edema, and hives) during weeks 68–70 that resolved with supportive medication and temporary interruption of the olipudase alfa infusions (completed).

There were no clinically significant abnormalities in laboratory findings, vital signs, electrocardiograms, or echocardiograms during the second year of treatment.

### Clinical outcomes

#### Spleen and liver volume

**Fig. 3:**
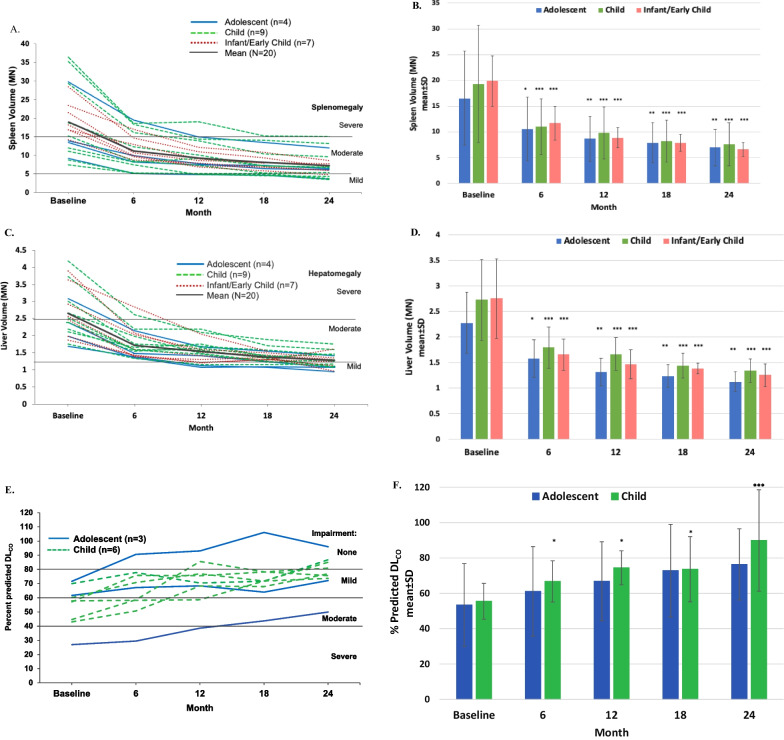
Changes in organomegaly and diffusing capacity of the lung over time with olipudase alfa treatment. **A** Individual patient responses for spleen volumes in multiples of normal (MN). Means for the overall population are indicated by the solid black line. Spleen absolute volumes were calculated as MN assuming normal volumes of 0.2% of body weight [17]. Severe and moderate splenomegaly were defined as > 15 and > 5 to ≤ 15 MN, respectively [17]. Cutoffs of MN for severity of splenomegaly are indicated by horizontal lines. **B** Mean spleen volumes in MN ± standard deviations over time stratified by baseline age group. *P* values from the ANCOVA test used to examine mean percent difference from baseline to month 24 are: *P* = 0.0014 for the adolescent group (n = 4) and *P* < 0.0001 for both the child (n = 7) and the infant/early child (n = 7) groups. Level of significance at each timepoint is indicated in the figure with asterisks (*p < 0.05; **p < 0.01; ***p < 0.001). Summary data at month 24 are provided in Additional file 1: Table S2. **C** Individual patient responses for liver volumes in MN. Means for the overall population are indicated by the solid black line. Liver absolute volumes were calculated as MN assuming normal liver volumes of 2.5% of body weight [17]. Severe and moderate hepatomegaly were defined as > 2.5 and > 1.25 to ≤ 2.5MN, respectively [17]. Cutoffs of MN for severity of hepatomegaly are indicated by horizontal lines. **D** Mean liver volumes in MN ± standard deviations over time stratified by baseline age group. P-values from the ANCOVA test used to examine mean percent difference from baseline to month 24 are: *P* = 0.0014 for the adolescent group (n = 4), *P* < 0.0001 for the child (n = 9), and *P* = 0.0002 for the infant/early child group (n = 6). Level of significance at each timepoint is indicated in the figure with asterisks (*p < 0.05; **p < 0.01; ***p < 0.001). Summary data at month 24 are provided in Additional file 1: Table S2. **E** Individual patient responses for percent predicted DL_CO_ adjusted for hemoglobin for patients able to perform the assessment at baseline. Cutoffs for gas exchange impairment are indicated (> 80% was considered normal/no impairment, > 60–80% mild impairment, 40–60% moderate impairment, and < 40% severe impairment) [19]. **F** Mean percent predicted DL_CO_ adjusted for hemoglobin ± standard deviations over time stratified by baseline age group. P-values from the ANCOVA test used to examine mean percent difference from baseline to month 24 are: *P* = 0.1747 for the adolescent group (n = 3) and *P* = 0.0002 for the child group (n = 6). Level of significance at each timepoint is indicated in the figure with asterisks (*p < 0.05; **p < 0.01; ***p < 0.001). Summary data at month 24 are provided in Additional file 1: Table S2

Splenomegaly and hepatomegaly were moderate or severe [17] at baseline and improved in all patients, with the largest magnitude of improvement during the first 6 months of treatment [14] followed by further incremental improvement over the subsequent 18 months (Fig. 3A, B for spleen and 3C and D for liver). Mean spleen volume decreased from 19.0 ± 8.8 MN at baseline to 7.2 ± 3.3 MN at 24 months (LS mean percent difference ± SEM − 60.9 ± 1.9%, *p* < 0.0001). Individual decreases in spleen volume ranged from 41.6 to 76.2%. All patients achieved spleen volumes in the mild to moderate [17] range by 24 months except for one patient with severe baseline splenomegaly whose 24-month value was borderline moderate at 15.1 MN. Decreases in spleen volume over time were similar across the different pediatric age groups (Fig. 3B).

Similar patterns were observed for hepatomegaly (Fig. 3C). Mean liver volume decreased from 2.7 ± 0.7 MN at baseline to 1.3 ± 0.2 MN at 24 months (LS mean percent difference ± SEM − 49.0 ± 1.7%, nominal *p* < 0.0001), with individual decreases ranging from 30.5 to 66.5%. All patients achieved liver volumes in the mild to moderate range [17] by 24 months. Decreases in liver volume over time were similar across the different pediatric age groups (Fig. 3D).

Summary data for mean decreases in organomegaly over time and percent change from baseline are provided in Additional file 1: Table S2.

#### Pulmonary disease

Among nine patients ≥ 5 years of age able to perform lung function tests, baseline percent predicted DL_CO_ was severely impaired in one patient, moderately impaired in four, and mildly impaired in four (Fig. 3E) [14]. Mean baseline percent predicted DL_CO_ was 54.8 ± 14.2 (moderate impairment [19]). DL_CO_ improved in all nine patients with baseline data by 24 months, with a LS mean percent difference ± SEM of 46.4 ± 4.1% (*p* = 0.0039). Individual changes from baseline ranged from 17% to 85.3%, with eight patients improving to no or mild impairment at 24 months, and one adolescent patient improving from severe impairment at baseline to moderate at 24 months (Fig. 3E). Mean percent predicted DL_CO_ for 14 patients with data at 24 months was 82.8 ± 27.5 (no impairment [19]). Mean percent predicted DL_CO_ stratified by age category showed similar improvements over time in both the child and adolescent groups (Fig. 3F). Summary data are provided in Additional file 1: Table S2.

Lung volumetric tests included forced vital capacity (FVC) and total lung capacity (TLC) among those able to perform lung function tests. The mean percent predicted values over time are shown in Additional file 1: Fig. S1. The mean percent predicted value improved from 77.4 ± 16.3% at baseline to 89.6 ± 23.4% at 24 months (LS mean difference ± SEM 22.6 ± 6.2%, *p* = 0.0081) for FVC and from 86.8 ± 23.3% to 122.6 ± 22.8% (LS mean difference ± SEM 52.9 ± 7.1, *p* = 0.002) for TLC.

**Fig. 4:**
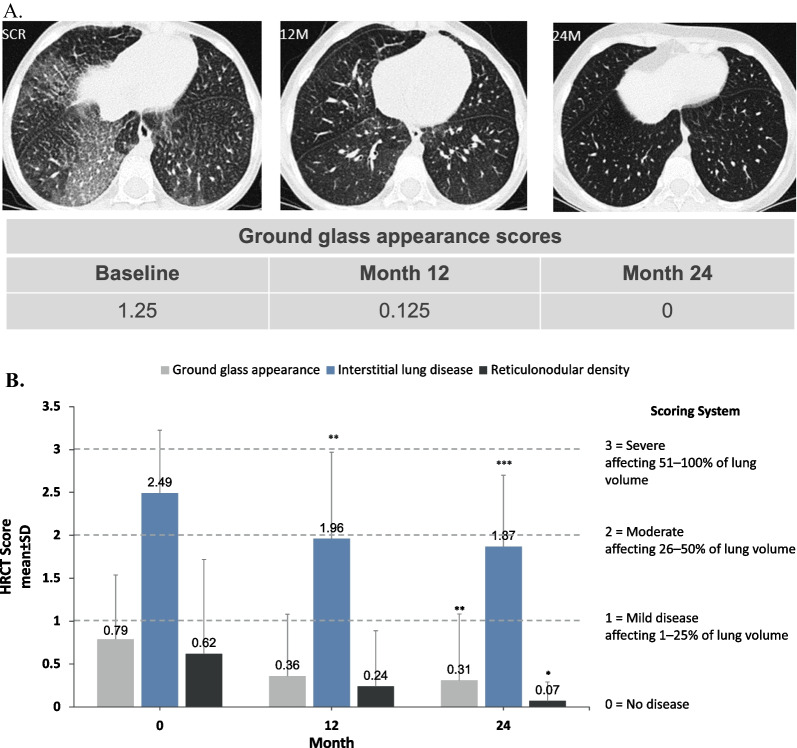
Changes in interstitial lung disease parameters over time with olipudase alfa treatment. **A** High-resolution computed tomography (HRCT) lung images of lower lung zones for a patient in the child group at baseline, 12 months, and 24 months. Scoring of images for ground glass appearance shown in the table below the images is based on a 4-point scale (0 = no disease; 1 = mild [1–25% lung volume affected]; 2 = moderate [26–50%]; 3 = severe [51–100%]). **B** Bar graph shows mean scores for ground glass appearance, interstitial lung disease, and reticulonodular density determined from HRCT images based on the percent of lung volume affected averaged over all four lung levels and both lungs. Images were scored based on the 4-point scale shown. Level of significance at each timepoint is indicated in the figure with asterisks (*p < 0.05; **p < 0.01; ***p < 0.001). Mean change from baseline to month 24 and *P* values are provided in the results section

As previously described [14], pulmonary imaging of lung regions was scored qualitatively by central readers for ground glass appearance, ILD, and reticulonodular density based percent of lung volume affected, where 0 = no disease; 1 = mild (≤ 25% of lung volume); 2 = moderate (26–50%); 3 = severe (51–100%). HRCT images and scores for ground glass appearance at baseline, 12 months and 24 months are shown in Fig. 4A for the patient (baseline age 7 years) who had the worst ground glass score at baseline. In this patient, ground glass opacity resolved with a score of 0 at 24 months.

At baseline, ground glass appearance scores were moderate to severe and ILD and reticulonodular density scores were mild. All three mean scores improved with 12 months of olipudase alfa treatment [14], and these improvements were maintained or further increased after 24 months (Fig. 4B). At 24 months, mean ± SD ground glass appearance score decreased from baseline by 0.44 points from 0.79 ± 0.75 to 0.31 ± 0.77 at 24 months (*p* = 0.0012), mean ILD score decreased by 0.70 ± 0.63 from 2.49 ± 0.74 to 1.87 ± 0.83 (*p* = 0.0001), and mean reticulonodular density score decreased by 0.58 ± 1.18 from 0.62 ± 1.1 to 0.07 ± 0.22 (*p* = 0.0463).

#### Growth

**Fig. 5:**
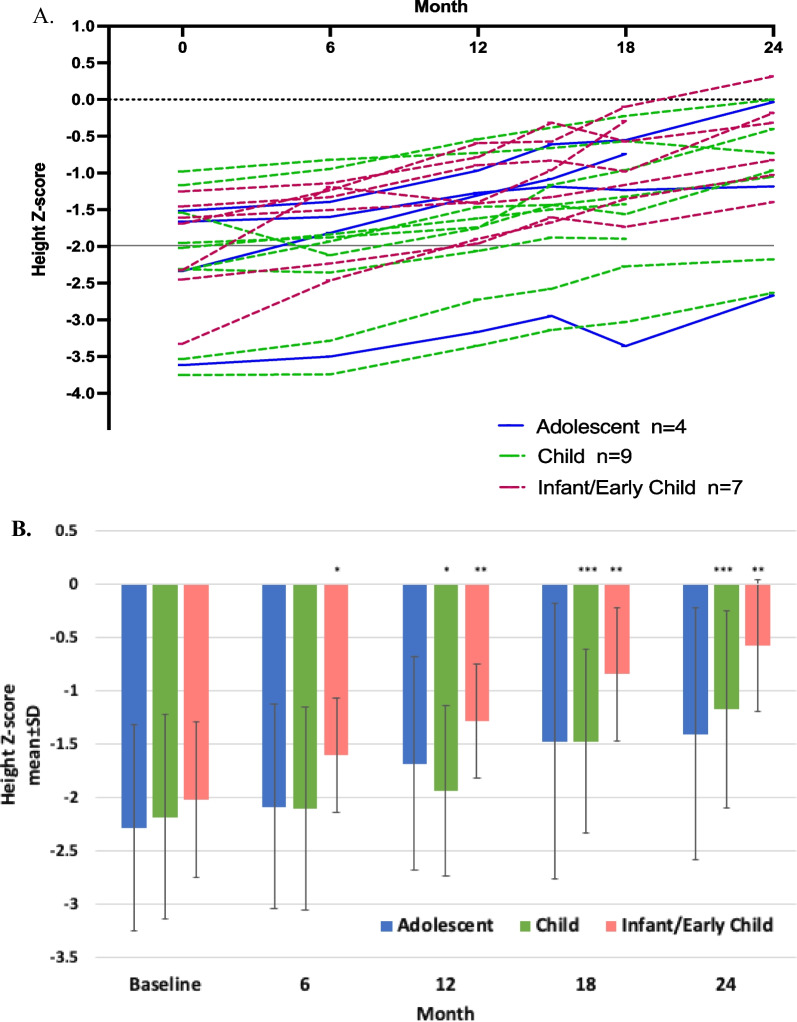
Changes in height Z-scores over time with olipudase alfa treatment. **A** Individual height Z-scores over time are shown. Per enrollment criteria, all patients were required to have a height Z-score of − 1.0 or less at baseline. The solid line at Z-score of − 2 indicates cutoff for short stature (height 2 or more standard deviations below the mean for age [20], equating to a Z-score of − 2 or less). A Z-score of 0 represents mean height. **B** Mean height Z-scores ± standard deviations over time stratified by age category. *P* values from the ANCOVA test used to examine mean difference from baseline to month 24 are: *P* = 0.2028 for the adolescent group (n = 3), *P* = 0.0005 for the child (n = 7), and *P* = 0.0016 for the infant/early child group (n = 6). Level of significance at each timepoint is indicated in the figure with asterisks (*p < 0.05; **p < 0.01; ***p < 0.001). Summary data at 24 months are provided in Additional file 1: Table S2

Individual height Z-scores over time are shown in Fig. 5A. Baseline height Z-scores ranged from − 3.8 to − 1.0 with a median of − 2.0 (mean − 2.1 ± 0.8). Ten patients (50%) met the criteria for short stature (i.e., height 2 or more standard deviations below the mean for age [20], equating to a Z-score of − 2 or less). Mean height Z-score improved from − 2.14 ± 0.84 at baseline to − 0.99 ± 0.88 at 24 months (LS mean difference ± SEM 1.17 ± 0.12, p < 0.0001) with individual scores ranging from − 2.7 to 0.3 (median − 0.9; mean − 1.0 ± 0.88). The proportion of patients with short stature at 24 months was 3/16 (19%). Mean height Z-score over time stratified by age category showed similar improvements over time (Fig. 4B).

For 19 patients with baseline and 24-month bone age data determined from wrist x-rays, the mean delay in bone age decreased from 25.6 ± 18.1 months to 17.0 ± 16.0 months after 2 years of olipudase alfa treatment (LS mean difference ± SEM 8.6 ± 2.8 months, *p* = 0.0065).

#### Other assessments

Improvements in liver function tests and plasma lipid profiles observed at 12 months[14] were maintained or further improved at 24 months (Additional file 1: Figs. S2 and S3, respectively), with mean values maintained within normal ranges. Mean baseline platelet counts reflected mild thrombocytopenia [14] and were improved at 24 months by 35.9 ± 32.4 × 10^9^/L (*p* = 0.0014) from 137.7 ± 62.3 × 10^9^/L to 166.9 ± 64.9 × 10^9^/L.

Both plasma lyso-sphingomyelin, the deacylated form of sphingomyelin and plasma chitotriosidase activity, a biomarker of activated macrophages, were elevated at baseline (mean values > 60 and > 6 times the upper limit of normal [ULN], respectively) and decreases reported by 6 months were maintained at 12 months [14]. At 24 months there was a mean reduction of pre-infusion plasma lyso-sphingomyelin of 87% and levels were near the ULN (Additional file 1: Fig. S4A). Plasma chitotriosidase activity was also close to the ULN at 24 months, reflecting a 76% reduction from baseline (Additional file 1: Fig. S4B).

## Discussion

In the absence of disease-specific treatment, natural history studies indicate that disease burden and mortality risk increase over time in children and adolescents with chronic forms of ASMD [4, 9, 21–23], with liver and lung disease [24] identified as major contributors to early death [7, 8]. The 2-year results of this trial demonstrate that a disease-modifying therapy such as olipudase alfa can substantially alleviate the disease burden in children from infancy through adolescence with chronic forms of ASMD.

Step-wise dose escalation is used for patients in olipudase alfa clinical trials [12, 14] to minimize adverse events arising from rapid production of high concentrations of bioactive catabolites and depletion of the substrate sphingomyelin [15, 16]. With this dose escalation regimen, metabolite levels and disease biomarkers decrease following treatment initiation, confirming olipudase alfa’s mechanism of action [12, 14]. Plasma levels of the sphingomyelin metabolite lyso-sphingomyelin levels shown to decrease over the first three months of olipudase alfa infusions in pediatric patients [14] remained stable at near normal values through 24 months of treatment. Activity of chitotriosidase, a biomarker of activated macrophages that was elevated at baseline decreased to near normal levels by 6 months [14], was stable through 24 months.

Olipudase alfa ERT was well tolerated over 24 months of treatment and there were no permanent discontinuations or study withdrawals due to adverse events. Most treatment-related events were mild to moderate IARs occurring primarily in the first 6 months of treatment and becoming less frequent over time, which is a typical observation with ERT [25]. IAR events occurring with olipudase alfa infusions were managed in some cases with temporary infusion interruptions or dose reductions that did not prevent reaching or maintaining the target olipudase alfa dose.

Olipudase alfa treatment resulted in significant improvements in key disease characteristics known to persist or worsen over time [9] in untreated pediatric patients of all ages with chronic ASMD. Deterioration in lung function occurs in children with untreated ASMD [9], and is associated with increased risk of respiratory infections and respiratory failure [6]. Progressive decline in pulmonary function also contributes to early mortality and decreased quality of life [8, 23, 26–28]. Lung function and ILD improved in all pediatric patients receiving olipudase alfa. Diffusing capacity of the lung assessed by determining the percent predicted DL_CO_ improved to mild or no impairment by 24 months in eight of nine children with baseline data, and to moderate impairment in one patient with severe impairment at baseline. Improvement in diffusing capacity was supported by pulmonary imaging assessments of ILD with decreases in mean scores for ground glass appearance, ILD, and reticulo nodular density.

A hallmark clinical manifestation of ASMD is moderate to severe organomegaly [8, 9, 29], which occurs early in life in children with chronic ASMD and does not resolve or improve over time [9]. Organomegaly in LSDs and other chronic diseases can impact satiety with consequences on nutrition and development in children [17, 30]. Splenomegaly in ASMD is also associated with increased bleeding risk [29]; thus, lifestyle modifications are often advised in order to reduce risk of splenic rupture [10]. All pediatric patients had reductions from baseline in spleen and liver volumes of greater than 30% by 24 months.

Other clinical parameters that worsen over time in pediatric patients with ASMD include dyslipidemia [4, 9] and thrombocytopenia [9]. Lipid profiles and platelet counts significantly improved over 12 months of olipudase alfa treatment [14] and showed additional improvements and normalization by 24 months of treatment. Elevated liver transaminases reflecting ASMD-associated liver pathology in pediatric patients [9, 29] normalized beginning in the first few weeks of olipudase alfa treatment [14] and remained within normal limits through 24 months.

Short stature is associated with poorer quality of life in children and adults [31]. Growth deficits in children and adolescents with chronic ASMD are common [9, 32, 33], although some patients achieve normal stature, often in association with homozygosity for the SMPD1 variant p.Arg610del [32, 34]. No patient enrolled in the ASCEND-Peds study was homozygous for p.Arg610del and all had growth deficits at baseline per inclusion criteria [14]. Improvements in Z-scores occurred in all patients, and the percentage of children with short stature decreased from 50% at baseline to 19% at 24 months of treatment. Mean bone age, which was delayed compared to actual age at the start of the study, increased over actual age for 19 children.

## Conclusion

ERT with olipudase alfa remained well-tolerated through 24 months of treatment in infants/young children, children, and adolescents with chronic ASMD, and treatment was associated with improvements in disease pathology across multiple clinically meaningful endpoints. Importantly, improvements observed during the first year of olipudase alfa treatment were maintained or further improved during the second year of treatment.

## Supplementary Information

**Additional file 1: Table S1**. Demographics and baseline characteristics. **Table S2**. Spleen and Liver Volumes, Percent Predicted DL_CO_, and height Z-scores at Baseline and 24 Months for by Age Group. **Fig. S1**. Results for volumetric lung function tests over time. Mean % predicted forced vital capacity (FVC) and total lung capacity (TLC) for overall pediatric population. **Fig. S2**. Plasma levels of liver enzymes and total bilirubin during treatment with olipudase alfa. **Fig. S3**. Plasma lipid concentrations during treatment with olipudase alfa. **Fig. S4**. Pre-infusion Plasma Levels Lyso-Sphingomyelin (**A**), and Chitotriosidase Activity (**B**) During Treatment with Olipudase Alfa.

## Data Availability

Qualified researchers may request access to patient level data and related study documents including the clinical study report, study protocol with any amendments, blank case report form, statistical analysis plan, and dataset specifications. Patient level data will be anonymized and study documents will be redacted to protect the privacy of trial participants. Further details on Sanofi's data sharing criteria, eligible studies, and process for requesting access can be found at: https://vivli.org.

## Abbreviations

ASM: Acid sphingomyelinase
ASMD: Acid sphingomyelinase deficiency
DL_CO_: Diffusing capacity of the lung for carbon monoxide
ERT: Enzyme replacement therapy
FVC: Forced fital capacity
HRCT: High-resolution computed tomography
IAR: Infusion associated reaction
ILD: Interstital lung disease
LS: Least square
MN: Multiples of normal
NPD: Niemann-Pick disease
SEM: Standard error of the mean
TLC: Total lung capacity
ULN: Upper limit of normal

## References

[1] Schuchman EH, Desnick RJ (2017). Types A and B Niemann–Pick disease. Mol Genet Metab.

[2] Bernadette B, Konrad S (2019). Lysosomal glycosphingolipid storage diseases. Ann Rev Biochem.

[3] Wasserstein MP, Desnick RJ, Schuchman EH, Hossain S, Wallenstein S, Lamm C (2004). The natural history of type B Niemann–Pick disease: results from a 10-year longitudinal study. Pediatrics.

[4] McGovern MM, Pohl-Worgall T, Deckelbaum RJ, Simpson W, Mendelson D, Desnick RJ (2004). Lipid abnormalities in children with types A and B Niemann Pick disease. J Pediatr.

[5] McGovern MM, Aron A, Brodie SE, Desnick RJ, Wasserstein MP (2006). Natural history of Type A Niemann–Pick disease: possible endpoints for therapeutic trials. Neurology.

[6] Cassiman D, Packman S, Bembi B, Turkia HB, Al-Sayed M, Schiff M (2016). Cause of death in patients with chronic visceral and chronic neurovisceral acid sphingomyelinase deficiency (Niemann–Pick disease type B and B variant): literature review and report of new cases. Mol Genet Metab.

[7] Barczykowski AL, Foss AH, Duffner PK, Yan L, Carter RL (2012). Death rates in the U.S. due to Krabbe disease and related leukodystrophy and lysosomal storage diseases. Am J Med Genet A.

[8] McGovern MM, Lippa N, Bagiella E, Schuchman EH, Desnick RJ, Wasserstein MP (2013). Morbidity and mortality in type B Niemann–Pick disease. Genet Med.

[9] McGovern MM, Wasserstein MP, Bembi B, Giugliani R, Mengel KE, Vanier MT (2021). Prospective study of the natural history of chronic acid sphingomyelinase deficiency in children and adults: eleven years of observation. Orphanet J Rare Dis.

[10] Wasserstein M, Dionisi-Vici C, Giugliani R, Hwu WL, Lidove O, Lukacs Z (2019). Recommendations for clinical monitoring of patients with acid sphingomyelinase deficiency (ASMD). Mol Genet Metab.

[11] Wasserstein MP, Diaz GA, Lachmann RH, Jouvin MH, Nandy I, Ji AJ (2018). Olipudase alfa for treatment of acid sphingomyelinase deficiency (ASMD): safety and efficacy in adults treated for 30 months. J Inherit Metab Dis.

[12] Wasserstein MP, Jones SA, Soran H, Diaz GA, Lippa N, Thurberg BL (2015). Successful within-patient dose escalation of olipudase alfa in acid sphingomyelinase deficiency. Mol Genet Metab.

[13] Wasserstein M, Lachmann R, Hollak C, Arash-Kaps L, Barbato A, Gallagher RC, et al. A randomized, placebo-controlled clinical trial evaluating olipudase alfa enzyme replacement therapy for chronic acid sphingomyelinase deficiency (ASMD) in adults: One-year results. Genet Med. 2022. 10.1016/j.gim.2022.03.021 35471153

[14] Diaz GA, Jones SA, Scarpa M, Mengel KE, Giugliani R, Guffon N (2021). One-year results of a clinical trial of olipudase alfa enzyme replacement therapy in pediatric patients with acid sphingomyelinase deficiency. Genet Med.

[15] McGovern M, Wasserstein M, Kirmse B, Duvall W, Schiano T, Thurberg B (2016). Novel first-dose adverse drug reactions during a Phase 1 trial of recombinant human acid sphingomyelinase (rhASM) in adults with Niemann–Pick disease type B (acid sphingomyelinase deficiency). Genet Med.

[16] Murray JM, Thompson AM, Vitsky A, Hawes M, Chuang W-L, Pacheco J (2014). Nonclinical safety assessment of recombinant human acid sphingomyelinase (rhASM) for the treatment of acid sphingomyelinase deficiency: the utility of animal models of disease in the toxicological evaluation of potential therapeutics. Mol Genet Metab.

[17] Pastores GM, Weinreb NJ, Aerts H, Andria G, Cox TM, Giralt M (2004). Therapeutic goals in the treatment of Gaucher disease. Semin Hematol.

[18] Greulich WW, Pule S. Radiographic atlas of skeletal development of the hand and wrist: Stanford University Press; 1959.

[19] Pellegrino R, Viegi G, Brusasco V, Crapo RO, Burgos F, Casaburi R (2005). Interpretative strategies for lung function tests. Eur Respir J.

[20] Barstow C, Rerucha C (2015). Evaluation of short and tall stature in children. Am Fam Physician.

[21] McGovern MM, Avetisyan R, Sanson BJ, Lidove O (2017). Disease manifestations and burden of illness in patients with acid sphingomyelinase deficiency (ASMD). Orphanet J Rare Dis.

[22] Mendelson DS, Wasserstein MP, Desnick RJ, Glass R, Simpson W, Skloot G (2006). Type B Niemann–Pick disease: findings at chest radiography, thin-section CT, and pulmonary function testing. Radiology.

[23] von Ranke FM, Pereira Freitas HM, Mancano AD, Rodrigues RS, Hochhegger B, Escuissato D (2016). Pulmonary involvement in Niemann–Pick disease: a state-of-the-art review. Lung.

[24] Labrune P, Bedossa P, Huguet P, Roset F, Vanier MT, Odievre M (1991). Fatal liver failure in two children with Niemann–Pick disease type B. J Pediatr Gastroenterol Nutr.

[25] Richards SM (2002). Immunologic considerations for enzyme replacement therapy in the treatment of lysosomal storage disorders. Clin Appl Immunol Rev.

[26] Freitas HMP, Mancano AD, Rodrigues RS, Hochhegger B, Torres P, Escuissato D (2017). Niemann–Pick disease type B: HRCT assessment of pulmonary involvement. J Bras Pneumol.

[27] Iaselli F, Rea G, Cappabianca S, Fabozzi G, Montemarano M, Vitale C (2011). Adult-onset pulmonary involvement in Niemann–Pick disease type B. Monaldi Arch Chest Dis.

[28] Hollak CE, de Sonnaville ES, Cassiman D, Linthorst GE, Groener JE, Morava E (2012). Acid sphingomyelinase (Asm) deficiency patients in The Netherlands and Belgium: disease spectrum and natural course in attenuated patients. Mol Genet Metab.

[29] McGovern MM, Wasserstein MP, Giugliani R, Bembi B, Vanier MT, Mengel E (2008). A prospective, cross-sectional survey study of the natural history of Niemann–Pick disease type B. Pediatrics.

[30] Larson-Nath C, Goday P (2019). Malnutrition in children with chronic disease. Nutr Clin Pract.

[31] Backeljauw P, Cappa M, Kiess W, Law L, Cookson C, Sert C (2021). Impact of short stature on quality of life: a systematic literature review. Growth Horm IGF Res.

[32] Wasserstein MP, Larkin AE, Glass RB, Schuchman EH, Desnick RJ, McGovern MM (2003). Growth restriction in children with type B Niemann–Pick disease. J Pediatr.

[33] Cox GF, Clarke LA, Giugliani R, McGovern MM (2018). Burden of illness in acid sphingomyelinase deficiency: a retrospective chart review of 100 patients. JIMD Rep.

[34] Lipinski P, Kuchar L, Zakharova EY, Baydakova GV, Lugowska A, Tylki-Szymanska A (2019). Chronic visceral acid sphingomyelinase deficiency (Niemann–Pick disease type B) in 16 Polish patients: long-term follow-up. Orphanet J Rare Dis.

