# Correction : Long-term safety and clinical outcomes of olipudase alfa enzyme replacement therapy in pediatric patients with acid sphingomyelinase deficiency: two-year results

**DOI:** 10.1186/s13023-023-02647-z

**Published:** 2023-03-14

**Authors:** George A. Diaz, Roberto Giugliani, Nathalie Gufon, Simon A. Jones, Eugen Mengel, Maurizio Scarpa, Peter Witters, Abhimanyu Yarramaneni, Jing Li, Nicole M. Armstrong, Yong Kim, Catherine Ortemann-Renon, Monica Kumar

**Affiliations:** 1 Department of Genetics and Genomic Sciences, Icahn School of Medicine at Mount Sinai, 1 Gustave L. Levy Place, New York, NY 10029 USA; 2 Medical Genetics Service HCPA, Department of Genetics UFRGS, DASA and Casa Dos Raros, Porto Alegre, Brazil; 3 Reference Centre of Inherited Metabolic Disease in Femme Mère Enfant Hospital, Hospices Civils of Lyon, Lyon, France; 4 Manchester University National Health Service Trust, St Mary’s Hospital, Manchester, UK; 5 Institute of Clinical Science for Lysosomal Storage Disorders, SphinCS GmbH, Mainz, Germany; 6 University Hospital of Udine, Udine, Italy; 7 University Hospitals Leuven, Louvain, Belgium; 8 Sanofi, Bridgewater, NJ USA; 9 Sanofi, Cambridge, MA USA; 10 Sanofi, Paris, France

**Correction :  Orphanet Journal of Rare Diseases (2022) 17:437** **https://doi.org/10.1186/s13023-022-02587-0**

Following publication of the original article [1], we have been notified that Fig. 1 was published incorrectly. Correct Fig. 1 should be as per below:

**Fig. 1:**
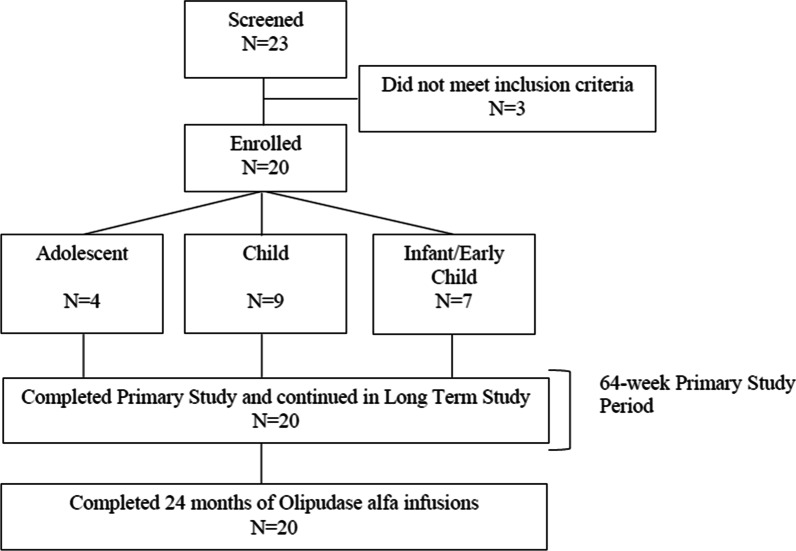
Patient disposition
