# Supplementary material for: Long-term safety and clinical outcomes of olipudase alfa enzyme replacement therapy in pediatric patients with acid sphingomyelinase deficiency: two-year results
Source: Orphanet J Rare Dis. 2022 Dec 14;17:437. doi: 10.1186/s13023-022-02587-0 (PMC9749157; doi:10.1186/s13023-022-02587-0)

Additional File 1.

Table S1: Demographics and Baseline Characteristics[14]

	**Adolescent (N = 4)**	**Child (N = 9)**	**Infant/Early Child (N = 7)**	**Overall (N = 20)**
Age at first infusion (years)				
Mean±SD	14.84±2.22	8.69±1.69	3.77±1.44	8.20±4.39
Median	14.8	8.72	3.75	7.99
Min : Max	12 :17	6 : 11.5	1 : 5.5	1.49 : 17.46
Sex, n %				
Male	3 (75)	4 (44)	3 (43)	10 (50)
Female	1 (25)	5 (56)	4 (57)	10 (50)
Race, n (%)				
White	3 (75)	7 (78)	7 (100)	17 (85)
Southeast Asian	1 (25)	1 (11)	0	2 (10)
Other	0	1 (11)	0	1 (5)
Ethnicity, n (%)				
Hispanic/Latino	0	0	1 (14)	1 (5)
Not Hispanic/Latino	4 (100)	9 (100)	6 (86)	19 (95)
Age at symptom onset (years)				
Mean±SD	1.370±0.581	1.633±1.295	1.170±0.903	1.420±1.017
Median	1.277	1.187	0.985	1.027
Min : Max	0.84 : 2.09	0.38 : 3.92	0.16 : 2.54	0.16 : 3.92
Age at Diagnosis (years)				
Mean±SD	2.124±0.708	3.358±3.360	1.551±1.200	2.478±2.449
Median	1.993	2.839	1.183	1.932
Min : Max	1.42 : 3.09	0.02 : 11.09	0.21 : 3.10	0.02 : 11.09
ASM activity (peripheral leukocytes) nmol/h/mg				
Mean±SD	0.210±0.092	0.129±0.061	0.095±0.067	0.135±0.078
Median	0.225	0.130	0.085	0.120
Min : Max	0.09 : 0.30	0.00 : 0.20	0.03 : 0.22	0.00 : 0.30
SMPD1 Variant, n (%)				
Homozygous for p.Arg610del	0	0	0	0
Heterozygous for p.Arg610del	1 (25.0)	3 (33.3)	2 (28.6)	6 (30.0)
Other variants	3 (75.0)	6 (66.7)	5 (71.4)	14 (70.0)
Full or partial splenectomy, n	0	0	0	0
Severe splenomegaly (>15 MN), n (%)	1 (25.0)	5 (55.6)	6 (85.7)	12 (60.0)
Severely reduced DLco (< 40%), n (%)	1 (33.3)	0	0	1 (11.1)[a]

[a] Based on nine patients able to perform the test at baseline

Table S2. Spleen and Liver Volumes, Percent Predicted DL_{CO}, and Height Z-scores at Baseline and Month 24 by Age Group. Percent Change from Baseline was calculated only for patients with values at both time points**.**

	**Age Group at Baseline**			
	**Adolescent**<br><br>**N=4**	**Child**<br><br>**N=9**	**Infant/early child**<br>**N=7**	**Overall**<br><br>**N=20**
**Spleen Volume MN**				
**Baseline**<br>mean±SD	16.6±9.1	19.3±11.4	19.9±4.9	19.0±8.8
**Month 24**	N=4	N=9	N=6	N=19
Observed mean±SD	7.0±3.6	7.6±4.2	6.6±1.4	7.2±3.3
% change from baseline LS mean±SEM	-57.4±2.1	-58.6±3.3	-66.8±2.3	-60.9±1.8
P value	0.0014	<.0001	<.0001	<.0001
**Liver Volume MN**				
**Baseline**<br>mean±SD	2.3±0.6	2.7±0.8	2.8±0.8	2.7±0.7
**Month 24**	N=4	N=9	N=6	N=19
Observed mean±SD	1.1±0.2	1.4±0.2	1.3±0.2	1.3±0.2
% change from baseline LS mean±SEM	-49.8±1.8	-48.4±2.6	-49.4±3.7	-49.0±1.7
P value	0.0014	<.0001	0.0002	<.0001
**Percent Predicted DL_{CO}**				
**Baseline**	N=3	N=6	0	N=9
mean±SD	53.4±23.4	55.5±10.1	NA	54.8±14.2
**Month 24**	N=4	N=9	N=1	N=14
Observed mean±SD	76.3±20.1	89.9±28.7	45.1	82.8±27.5
% change from baseline LS mean±SEM	45.4±12.8	47.2±3.7	NA	46.6±25.5
P value	0.1747	0.0002		<.0001
**Height Z-scores**				
**Baseline**	N=4	N=9	N=7	N=20
mean±SD	-2.3±1.0	-2.2±1.0	-2.0±0.7	-2.1±0.8
**Month 24**	N=3	N=7	N=6	N=16
Observed mean±SD	-1.4±1.2	-1.2±0.9	-0.6±0.6	-1.0±0.9

change from baseline LS mean±SEM	0.9±0.3	1.0±0.1	1.5±0.2	1.2±0.1
P value	0.2028	0.0005	0.0016	<.0001

LS least square; MN multiples of normal

Figure S1.
Results for volumetric lung function tests over time. Mean % predicted forced vital capacity (FVC) and total lung capacity (TLC) for overall pediatric population.

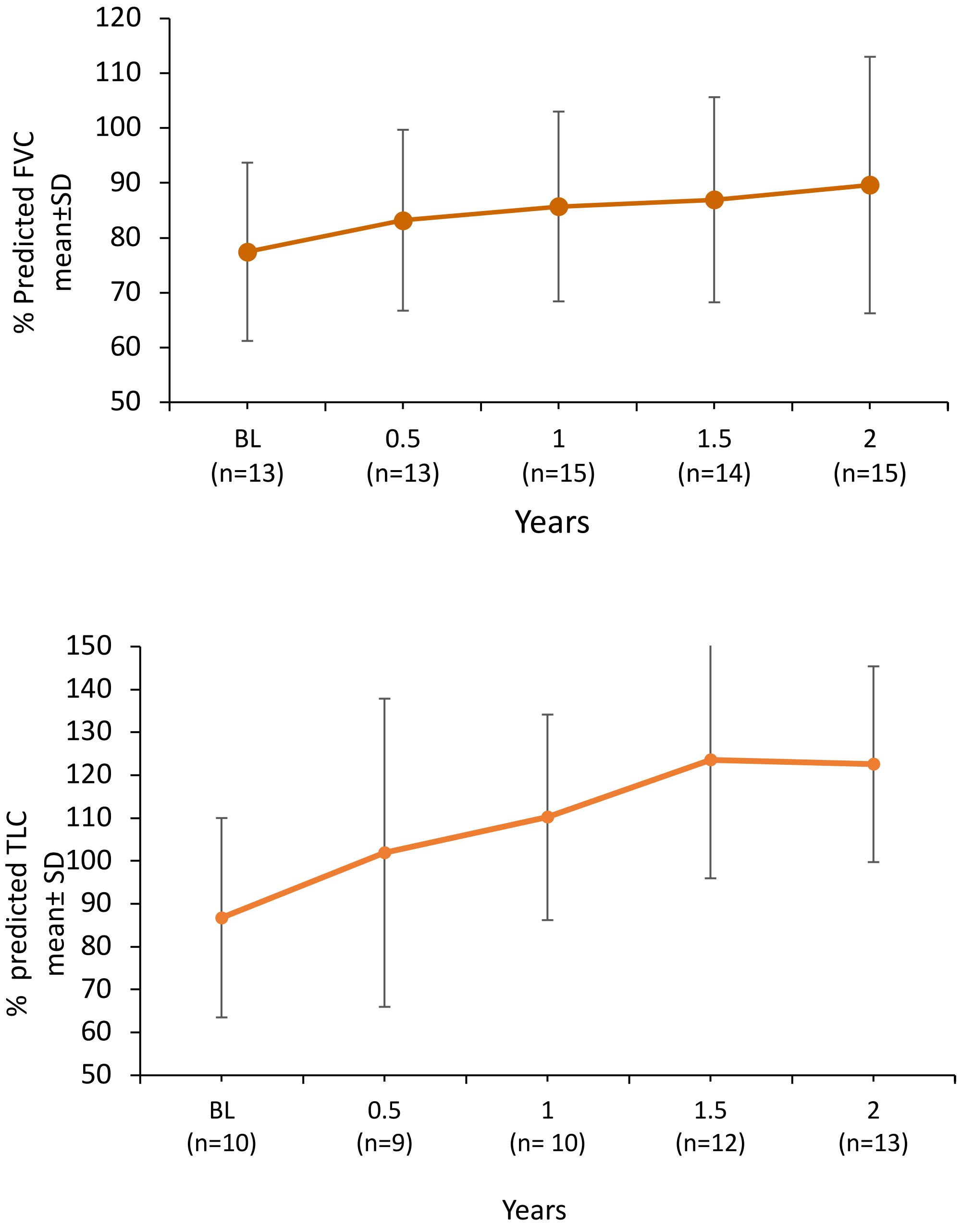

Figure S2. Plasma levels of liver enzymes and total bilirubin during treatment with olipudase alfa

Mean±SD pre-infusion plasma levels for aspartate aminotransferase (AST), alanine aminotransferase (ALT) and total bilirubin for all pediatric patients. The AST and ALT upper limit of normal (ULN) for the Adolescent, Child and Infant/Early Child groups is 40, 59 and 69 IU/L and 43, 34, and 34 IU/L, respectively. Mean total bilirubin levels were within normal ranges at baseline and throughout the study and all patients had normal levels at 24 months (ULN 20 µmol/L). The mean±SD percent decrease at 24 months was 64.4±16.6 (nominal $p<0.0001$), 56.0±14.5 (nominal $p<0.0001$) and 45.3±18.2 (nominal $p<0.0001$) for ALT, AST, and total bilirubin, respectively.

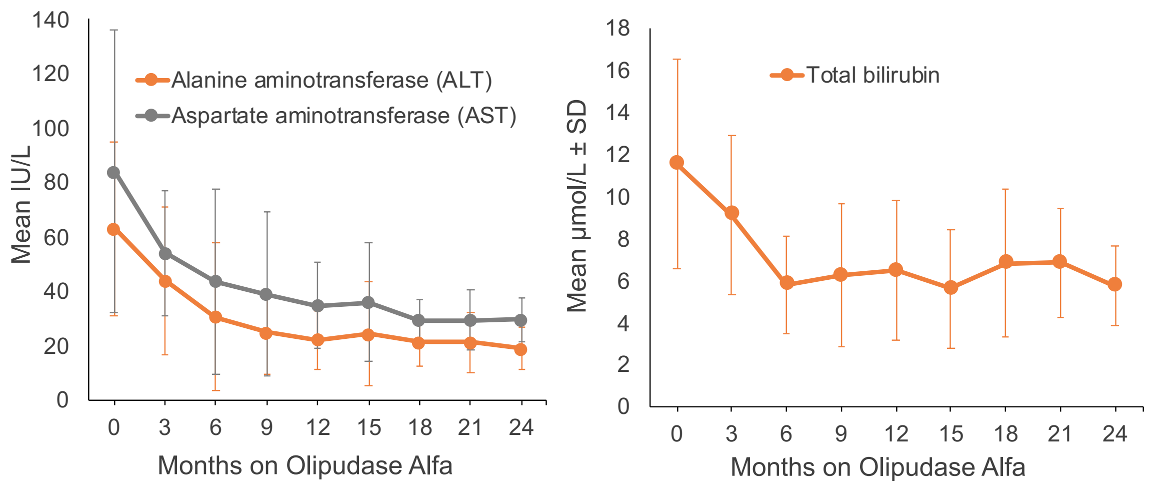

Figure S3. Plasma lipid concentrations during treatment with olipudase alfa
Mean (SD) values for total cholesterol, low density lipid (LDL) cholesterol, high density lipid (HDL) cholesterol, and triglycerides for all pediatric patients. Upper limits of normal across age groups as available are indicated by horizontal lines. The percent change in mean concentrations at month 24 are indicated above each graph.

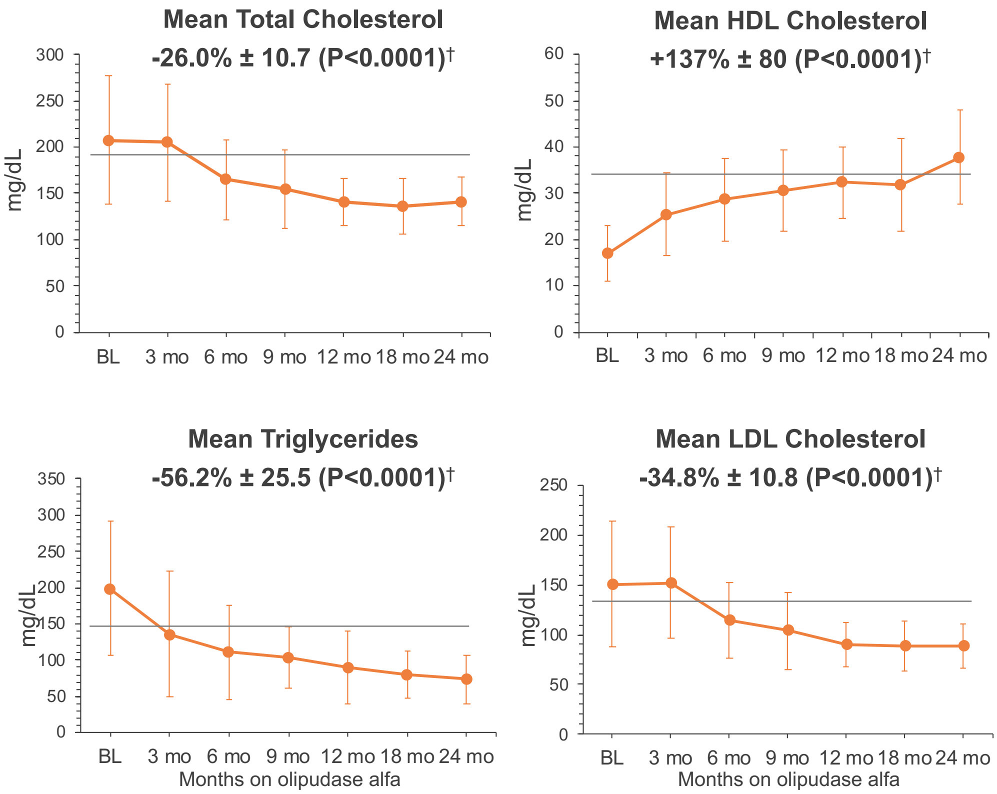

†All p values nominal

Figure S4. Pre-infusion Plasma Levels Lyso-Sphingomyelin (A), and Chitotriosidase Activity (B) During Treatment with Olipudase Alfa

Chitotriosidase data were normalized based on *CHIT1* genotype. Those homozygous for the null mutation were excluded and values for heterozygous patients were doubled.

A.

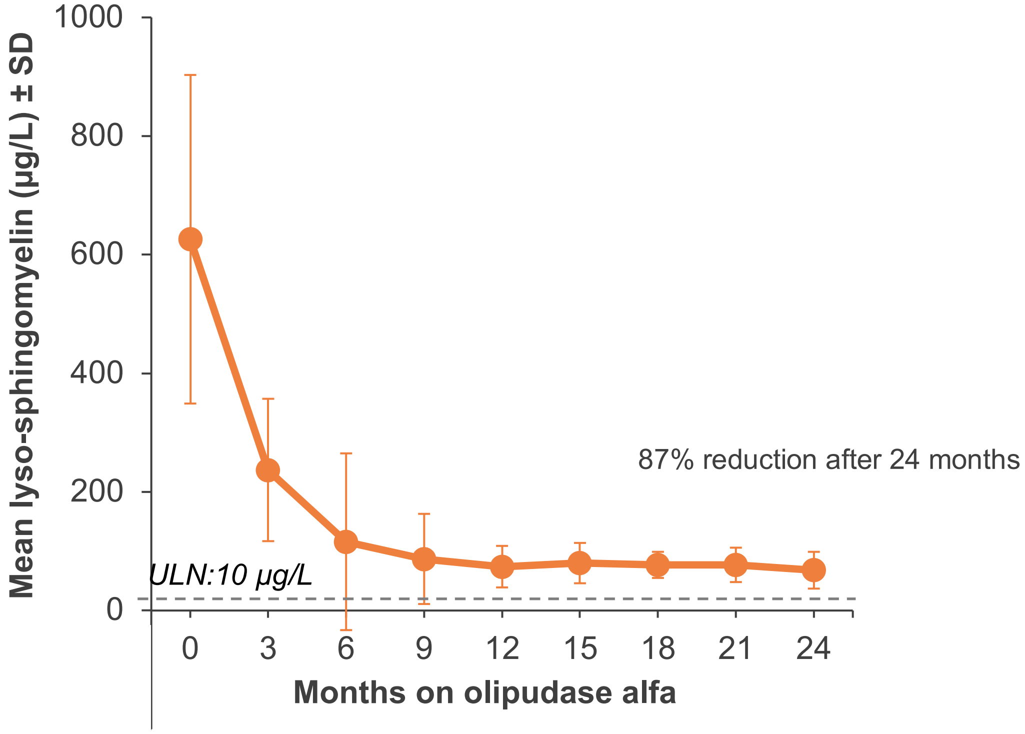

B.

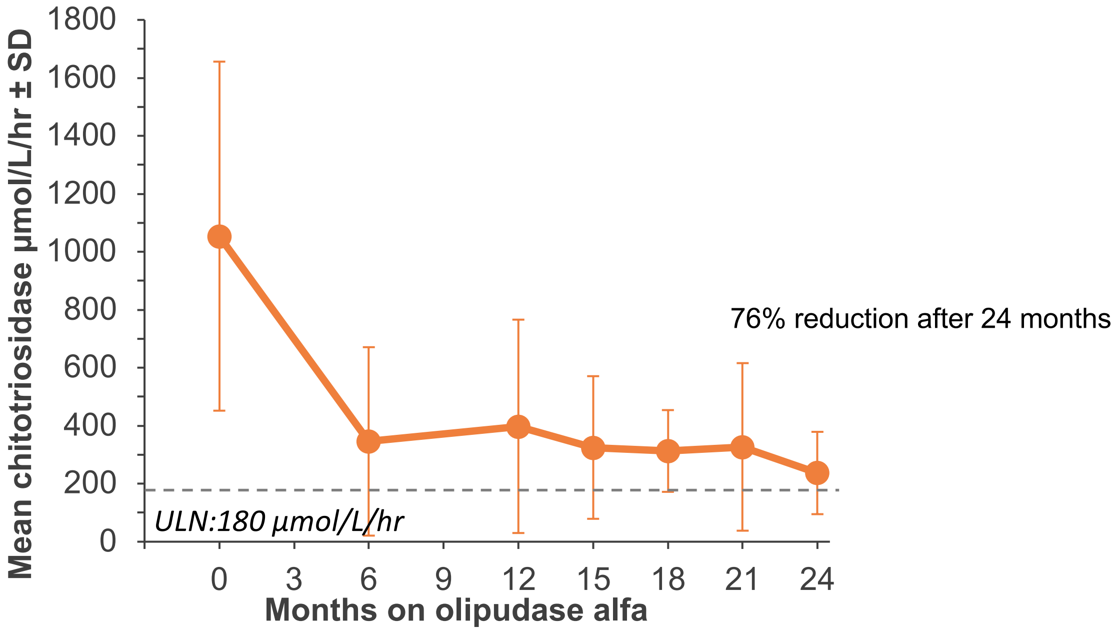

Supplement: Supplementary file 1 — Additional file 1: Table S1. Demographics and baseline characteristics. Table S2. Spleen and Liver Volumes, Percent Predicted DLCO, and height Z-scores at Baseline and 24 Months for by Age Group. Fig. S1. Results for volumetric lung function tests over time. Mean % predicted forced vital capacity (FVC) and total lung capacity (TLC) for overall pediatric population. Fig. S2. Plasma levels of liver enzymes and total bilirubin during treatment with olipudase alfa. Fig. S3. Plasma lipid concentrations during treatment with olipudase alfa. Fig. S4. Pre-infusion Plasma Levels Lyso-Sphingomyelin (A), and Chitotriosidase Activity (B) During Treatment with Olipudase Alfa. [file 13023_2022_2587_MOESM1_ESM.pdf]
